# The relationship between proteome size, structural disorder and organism complexity

**DOI:** 10.1186/gb-2011-12-12-r120

**Published:** 2011-12-19

**Authors:** Eva Schad, Peter Tompa, Hedi Hegyi

**Affiliations:** 1 Institute of Enzymology, Research Center For Natural Sciences, Hungarian Academy of Sciences, Karolina út 29. Budapest, 1113 Hungary

## Abstract

**Background:**

Sequencing the genomes of the first few eukaryotes created the impression that gene number shows no correlation with organism complexity, often referred to as the G-value paradox. Several attempts have previously been made to resolve this paradox, citing multifunctionality of proteins, alternative splicing, microRNAs or non-coding DNA. As intrinsic protein disorder has been linked with complex responses to environmental stimuli and communication between cells, an additional possibility is that structural disorder may effectively increase the complexity of species.

**Results:**

We revisited the G-value paradox by analyzing many new proteomes whose complexity measured with their number of distinct cell types is known. We found that complexity and proteome size measured by the total number of amino acids correlate significantly and have a power function relationship. We systematically analyzed numerous other features in relation to complexity in several organisms and tissues and found: the fraction of protein structural disorder increases significantly between prokaryotes and eukaryotes but does not further increase over the course of evolution; the number of predicted binding sites in disordered regions in a proteome increases with complexity; the fraction of protein disorder, predicted binding sites, alternative splicing and protein-protein interactions all increase with the complexity of human tissues.

**Conclusions:**

We conclude that complexity is a multi-parametric trait, determined by interaction potential, alternative splicing capacity, tissue-specific protein disorder and, above all, proteome size. The G-value paradox is only apparent when plants are grouped with metazoans, as they have a different relationship between complexity and proteome size.

## Introduction

Biological complexity is a feature that increases during evolution, distinguishing us from more primitive forms of life. Whereas it has no straightforward definition, it is generally accepted that it can be measured by the number of different cell types in an organism ranging from 1 (bacteria) to about 200 (humans) [1-4]. As complexity is apparently related to the amount of information an organism needs to function properly, and such information is contained in our genes, it was generally expected that the number of genes correlates with biological complexity. This was called into doubt and referred to as the G-value paradox [5]. There have been numerous attempts to resolve the paradox, citing multifunctionality of proteins [6], microRNAs [7], non-protein-coding DNA [8] or alternative splicing [9]. In this paper we set out to revisit this problem as the genomes of many more eukaryotes have been sequenced and new information has accumulated about their alternative splicing. In addition, we have paid special attention to the roles intrinsically disordered proteins (IDPs) might play in this respect in these organisms.

Intrinsically disordered proteins exist and function without a well-defined three-dimensional structure, typically carrying out signaling and regulatory functions [10,11]. These functions are linked with complex responses to environmental stimuli and communication between cells, which raises the question of whether structural disorder can be linked to the complexity of species. This view is underscored by structural disorder being critical in protein-protein interactions (PPIs) [12-14], in the assembly of large protein complexes [15], and multiple activities of proteins [16]. Compounded by the observation that the level of disorder is much higher in eukaryotes than prokaryotes [17], it is often implied that structural disorder increases with complexity [18,19].

Here we carried out a systematic analysis of the possible correlation between proteome size, structural disorder, binding capacity and the complexity of 76 organisms ranging from bacteria to human, which cover the full complexity range of 1 to 200. We used the number of amino acids instead of the number of genes, as average protein length tends to vary a great deal among different organisms (from 282 to 814 in our collection). However, we found no overall, proteome-wide correlation between protein structural disorder and organism complexity, apart from the clear increase in disorder from prokaryotes to eukaryotes, with very large variations between different bacteria and also single-celled eukaryotes - for example, protozoa. When we looked at only those proteins that comprised domains associated with evolutionary expansion [2], we found that such proteins were significantly more disordered than the rest of the proteomes.

We analyzed another structural disorder-related feature, namely binary interactions in interactomes [12-14], and predicted interaction capacity of proteins in their disordered regions [20]. We found that the total number of predicted binding sites correlated with the complexity of the organism but the average number of binding sites per protein did not. We extended these studies to human tissues, which are also thought to have different complexities. Analogously to the wide range of organisms appearing in this paper, we determined the complexity of human tissues as the number of different cell types they are composed of. We found significant differences in structural disorder and a clear-cut correlation with complexity of the tissue. We also analyzed protein binding sites and PPIs in the different human tissues and found a significant correlation between the two, following a power law distribution. The relationship was close to a quadratic one, signifying the prevalence of promiscuous, rather than one-on-one, protein binding. Alternative splicing also proved to be more prevalent in tissues that are regarded to be more complex and ranked similarly to other aspects, that is, disorder and protein binding.

Our overall conclusion is that complexity is a multi-parametric trait affected by interaction potential, alternative splicing capacity, tissue-specific protein disorder and, above all, proteome size, thus severely limiting the scope of the G-value paradox.

## Results

### (Dis)solving the G-value paradox

**Figure 1:**
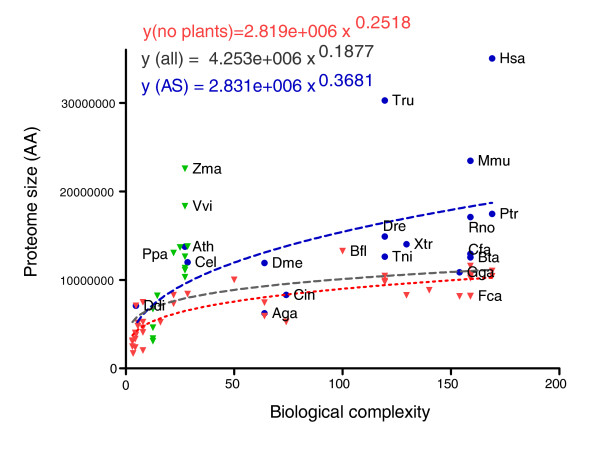
**Proteome size as a function of complexity**. Total numbers of amino acids (AA) are shown for each proteome as a function of the complexity of the organism, measured by the number of cell types the organism has. The total sum of the longest variants, presumably corresponding to the main isoforms, is shown with green (plants) and red (eukaryotes without plants) triangles whereas blue dots indicate the alternative complements where all the known variants for a gene are included. Both the main isoform proteomes and the alternative splice complements follow a power function, with an exponent of 0.25/0.19 (eukaryotes without and with plants) and 0.27, respectively. Abbreviated species names are shown for some plants and the alternative proteomes: Aga, *Anopheles gambiae*; Ath, *Arabidopsis thaliana*; Bfl, *Branchiostoma floridae*; Bta, *Bos taurus*; Cel, *Caenorhabditis elegans*; Cfa, *Canis familiaris*; Cin, *Ciona intestinalis*; Ddi, *Dictyostelium discoideum*; Dme, *Drosophila melanogaster*; Dre, *Danio rerio*; Fca, *Felis silvestris cattus*; Gga, *Gallus gallus*; Has, *Homo sapiens*; Mmu, *Mus musculus*; Rno, *Rattus norvegicus*; Ppa, *Physcomitrella patens*; Ptr, *Pan troglodytes*; Tni, *Tetraodon nigroviridis*; Tru, *Takifugu rubripes*; Vvi, *Vitis vinifera*; Xtr, *Xenopus tropicalis*; Zma, *Zea mays*. See Additional file 3 for the full list of proteomes.

The authors who coined the term 'G-value paradox' [5] questioned its validity themselves (using only eukaryotic organisms), referring instead to an I-value, that is, the information content of a genome, which, however, they did not quantify. In place of these two measurements we have used instead the total proteome information content (PIC), defining it here as the total number of amino acids in the longest isoform of each gene in an organism. Figure 1 shows the values for each eukaryote in our collection as the function of complexity measured by the number of cell types an organism has. The best-fitting function describing the relationship between cell types and PIC is a power function, with the exponent being around 0.25 if we exclude plants (approximately 0.19 with plants). This means that complexity is roughly proportional to the 4th (5th with plants) power of the PIC value contained in the proteome of an organism, that is, it grows more slowly than a linear function.

We did distinguish plants from other eukaryotes in Figure 1 because they clearly differ in their biological complexity-proteome size relationship from other evolutionary clades in that they have relatively large proteomes for a smaller number of cell types. While there is a weak but significant overall relationship between biological complexity and proteome size when all eukaryotic organisms are considered in Figure 1 (assuming a Gaussian distribution R^2 ^= 0.1333, *P*-value = 0.0072), if we leave out plants the correlation increases dramatically (R^2 ^= 0.6326, *P*-value < 0.0001).

We calculated the relationship also for the alternative splice complement of those organisms (Figure 1) for which there are data about their splice variants, taking into account all variants, and found the same type of relationship but a noisier one as alternative splicing information is scarce and much less reliable than the proteome of the main isoforms (R^2 ^= 0.262, *P*-value = 0.0357). Both human and fugu (Tru, *Takifugu rubripes*) have strikingly large alternative proteomes, although these are based only on RNA sequence information, which does not necessarily mean that viable proteins are produced from them [21].

We repeated the calculation using gene numbers in place of proteome sizes (Additional file 1) and found a weak but significant correlation between complexity and gene number only when we treated plants as a separate group; strictly speaking, therefore, the G-value paradox still holds up, but only when we consider plants together with other eukaryotes.

To make sure the results are not confounded by phylogenetic pseudoreplication [22] - that is, bias in statistics due to lack of statistical independence among closely related species we used in our studies - we grouped the 76 species into 6 large phylogenetic groups and used one-way analysis of variance (ANOVA) to see if they have significantly different values for the PIC proteome size (Additional file 2). We found, in accordance with the species-level studies, that (i) plants have larger proteomes than other metazoan groups, (ii) there is no significant difference in proteome size between fungi and protozoa, and (iii) all the other phylogenetic groups are significantly different from one another, increasing with complexity.

### Structural disorder

**Figure 2:**
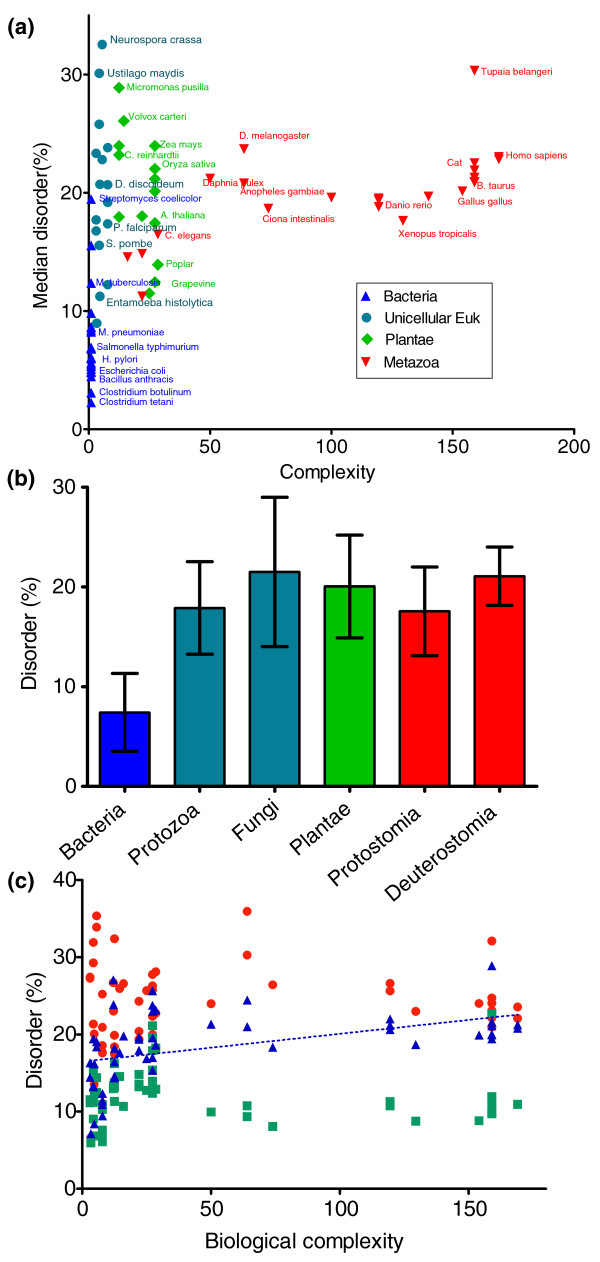
**Structural disorder of proteins in prokaryotic and eukaryotic species**. **(a) **Average structural disorder in prokaryotes and eukaryotes was predicted by the IUPred algorithm [25], averaged over all proteins in the proteome, and is shown as a function of biological complexity of the species. **(b) **Species were grouped into six clusters and average disorder within these groups was calculated (error bars represent standard deviation). **(c) **Proteins were sorted on the basis of their Structural Classification of Proteins (SCOP) [43] domain expansions and their average disorder is shown as a function of biological complexity of the species. In particular, there are proteins in which domain expansion correlates (red circle) or does not correlate (green square) with biological complexity. For comparison, proteins are also shown that contain any kind of SCOP domain (blue triangle).

The observation of the sharp increase in predicted disorder upon transition from prokaryotes to eukaryotes [23] has been taken to suggest correlation of structural disorder with complexity [17,24]. To check if this is true, we calculated the average disorder for the 76 proteomes in our collection (listed in Additional file 3) using the IUPred algorithm [25] and plotted it as a function of biological complexity, that is, the number of different cell types (Figure 2a). For the latter we used data taken from [1-4], and for bacteria we assumed a complexity of one.

Overall, there is correlation between the two measures only if we consider all species (Spearman correlation coefficient r = 0.6545, *P*-value < 0.0001). For eukaryotes only, there is no significant correlation between biological complexity and proteome disorder (r = 0.1284, *P*-value = 0.3594; there is also no correlation for eukaryotes even when we exclude plants, r = 0.2449, *P*-value = 0.1329) whether considering individual species or combining them into the same large evolutionary clades as in Additional file 2 - protozoa, fungi, plants, protostomes and deuterostomes (Figure 2b). Within bacteria, the overall disorder is usually low, in the range of 2 to 10%, with some exceptions, such as *Mycobacterium tuberculosis*, *Myxococcus xanthus *and *Streptomyces coelicolor*. The high disorder of *M. tuberculosis *may be a result of its high GC content and preference towards amino acids Ala, Gly, Pro, Arg and Trp [17] and/or the result of the lifestyle of the bacterium [26]. The disorder of protozoa and fungi shows large variations between 9% and 30%, spanning a range that covers disorder in all higher eukaryotes. In conclusion, these data show a significant difference between the intrinsic disorder of prokaryotes (complexity = 1) and eukaryotes only but no correlation with the complexity of large eukaryotic groups (for eukaryotes, complexity ranges from 3 to 169).

Next, we examined disorder in those groups of proteins where the evolutionary expansion of Structural Classification of Proteins (SCOP) domains correlated [2] with complexity (Pearson correlation coefficient > 0.8) and compared them to those that did not (correlation coefficient < 0.2), and also to the total sets of proteins that contained at least one SCOP domain of any kind. Average structural disorder was calculated for these three groups of proteins in every selected prokaryotic and eukaryotic proteome (Figure 2c; Additional file 4).

For eukaryotes, the average disorder of proteins with expanding type of SCOP domains (Figure 2c, red circles) is always higher than for proteins with domains of the non-expanding type (green squares). For prokaryotes, the number of expanding domains is too low to make a statistically meaningful comparison. The disorder of proteins that contain any-type SCOP domains was usually between these two values (Figure 2c, blue triangles). Because in eukaryotic genomes protein families with expanding SCOP domains are mostly involved in regulation or extracellular processes [2], the expansion of these domains is likely to have contributed to the emergence of new cell types and complex intercellular communication.

Regarding interspecies differences, we found again that apart from a significant jump in disorder between prokaryotes and eukaryotes, neither the expanding nor the non-expanding SCOP domain-containing proteins showed a significant correlation between complexity and disorder among the eukaryotic species. However, for the set of the any-type SCOP domain-containing proteins (blue triangles in Figure 2c) we did find a significant correlation between complexity and protein disorder (Spearman correlation r = 0.6597, *P *< 0.0001) among the eukaryotic species. This correlation may be caused by a lengthening of structural domain-containing proteins during evolution (Additional file 4) in concert with increased overall disorder, perhaps due to longer linker regions that might also increasingly serve as new disordered binding sites (see next section).

### Protein-protein interactions

Intrinsically disordered proteins often function via specific binding to other proteins, DNA or RNA [27]. The underlying process of coupled folding and binding have several advantages, such as the ability to bind multiple partners or uncoupling of specificity from binding strength, allowing weak but specific interactions. These features may also be directly linked with complexity because complexity intuitively may be related to the number of interactions proteins make, that is, the size of the interactome. This issue can be addressed in two different ways.

**Figure 3:**
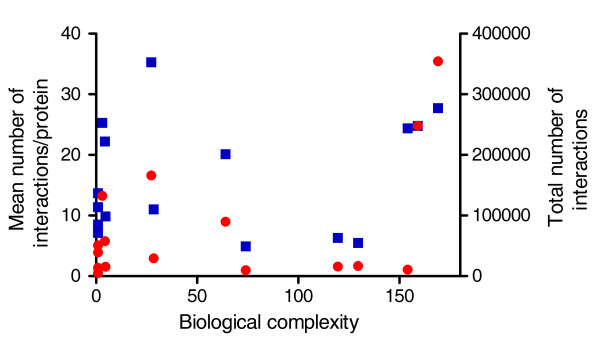
**Number of protein-protein interactions of prokaryotic and eukaryotic species**. The total number of binary interactions (red circles) and the number of high confidence (score ≥ 900) interactions per protein (blue squares) was calculated for 16 prokaryotic and eukaryotic proteomes (see the 'Protein-protein interactions in different proteomes' section in Materials and methods), and are shown as a function of biological complexity.

The direct way is to analyze the size of the interactomes of species of different complexity. We extracted binary interactions for the available prokaryotic and eukaryotic proteomes from the STRING database. The total number of binary interactions and the number of interactions per protein were calculated for each proteome and are shown for the high-confidence interaction data (confidence score ≥ 900 in STRING) as a function of biological complexity in Figure 3 (for all data, see Additional file 3). However, we found no correlation between either the total number of binary interactions or the number of interactions per protein and biological complexity. The most plausible reason is the insufficient coverage of the interactomes insofar as allowing genome-wide comparisons.

Because of these uncertainties, we used predictions to get an unbiased estimate of binding capacity of the different proteomes. We used ANCHOR, a method developed for the prediction of binding regions embedded in disordered regions [20]. We carried out ANCHOR predictions for our selected species, and express the results as the mean and total number of disordered binding sites (Additional file 3).

**Figure 4:**
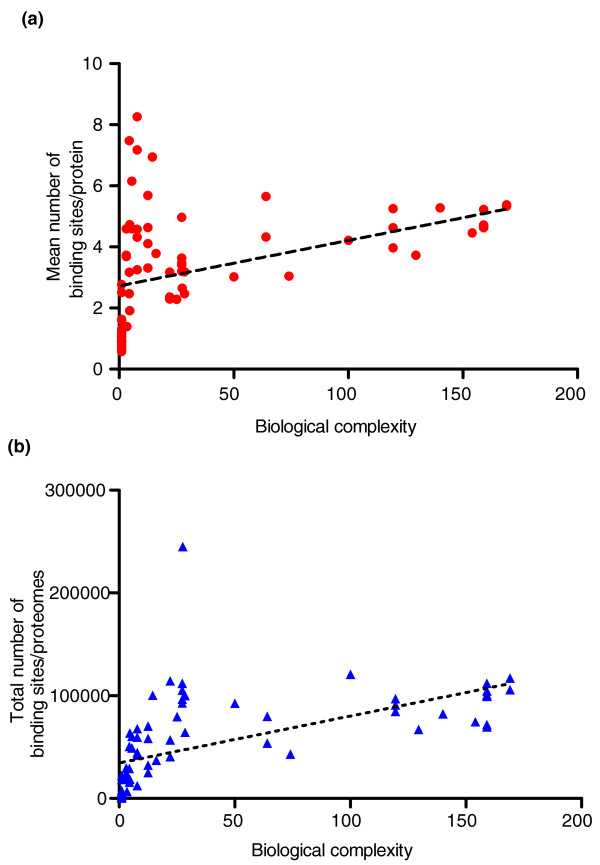
**Predicted binding sites of proteins in prokaryotic and eukaryotic species**. **(a) **The mean number of binding sites within disordered regions predicted by ANCHOR [20] in prokaryote and eukaryote species is shown as a function of biological complexity of the species. **(b) **The total number of binding sites predicted as in (a) per proteome is shown as a function of biological complexity of the species.

When all species are considered there is a highly significant correlation between the mean number of binding sites per protein and biological complexity (r = 0.7000, *P *< 0.0001; Figure 4a). For eukaryotes only, there is no significant correlation between biological complexity and the mean number of binding sites per protein (r = 0.2057, *P *= 0.1395); if we exclude plants, the correlation becomes significant but only marginally (r = 0.3441, *P *= 0.0319). However, the correlation between the total number of binding sites within entire proteomes and complexity (Figure 4b) is highly significant in the case of both all species (r = 0.8786, *P *< 0.0001) and eukaryotes only (r = 0.7079, *P *< 0.0001). These results suggest that an increase in the number of potential disordered binding sites and the concomitant increase in the size and complexity of the interactome have probably contributed to the increase in complexity in evolution.

### Alternative splicing

Alternative splicing increases proteome size and the complexity of the transcriptome without an increase in genome size by generating multiple mRNA products from a single gene. By conservative estimations, at least 40 to 60% of human and other mammalian genes undergo alternative splicing [28-30], with more recent studies putting the number even higher [31,32]. Less complex organisms have less alternative splicing, fission yeast having only a handful of known cases [33]. To put these inferences on quantitative ground, we studied the extent of alternative splicing among animals with distinct complexity using the ASAP II database [9].

**Figure 5:**
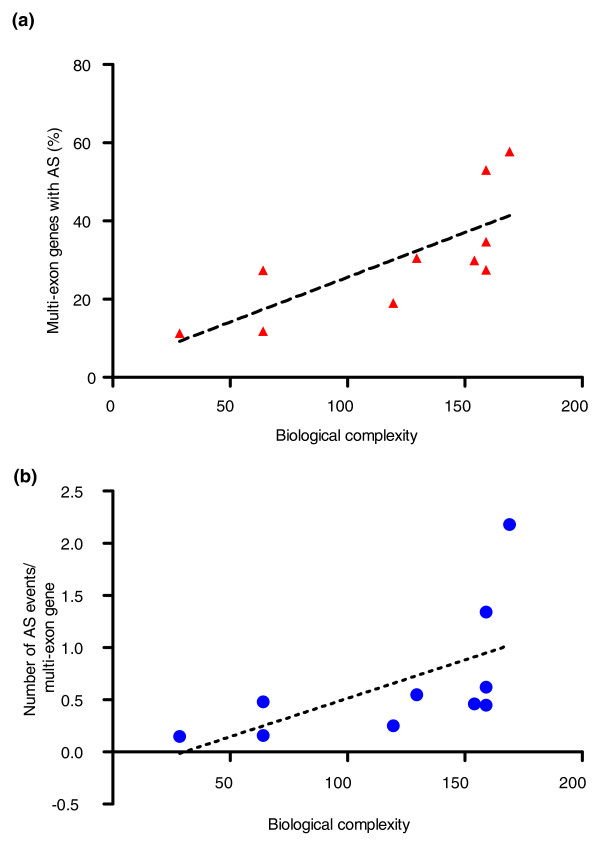
**Alternative splicing in some animal species**. **(a,b) **The percentage of multi-exon genes that have alternative splice (AS) forms (a) and the mean number of alternative splicing events per gene (b) were calculated for ten studied proteomes (see the 'Alternative splicing' section in Materials and methods) and are shown as a function of biological complexity.

We determined the percentage of multi-exon genes that have alternative splice forms (Figure 5a) and also the mean number of alternative splicing events per gene for each studied genome (Figure 5b). Both parameters correlate with biological complexity, with the percentage of multi-exon genes with alternative splicing being more significant (r = 0.8924, *P *= 0.0011) than the number of alternative splicing events per gene (r = 0.7816, *P *= 0.0105). Protein isoforms of more complex organisms also contain more exons (Additional file 3). These findings confirm that vertebrates (mammals in particular) have higher rates of alternative splicing than less complex organisms with a similar, or even higher, number of genes. The exact numbers should be handled carefully, though, because the analyses depend a lot on the expressed sequence tag coverage, which varies greatly between the organisms included.

### Complexity of tissues/organs

Alternative splicing, structural disorder and PPIs also tend to vary considerably among different tissues of the same organism, presumably reflecting the complexity of the tissue [34]. Comparing tissues instead of different organisms also has the advantage of more coherent data, not so dependent on the number of studies an organism has been subjected to.

**Figure 6:**
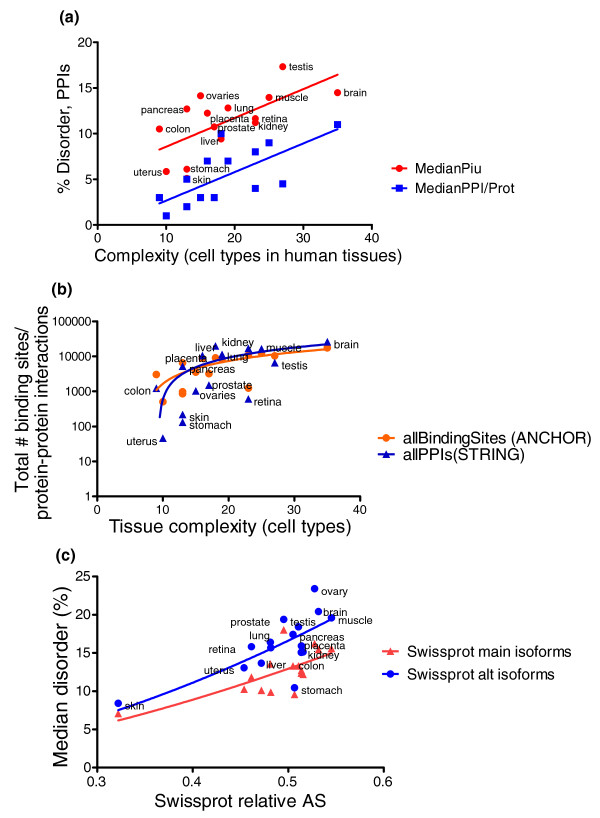
**Disorder, protein-protein interactions and binding of proteins expressed in different human tissues**. **(a) **Median disorder and median number of binding proteins, both as a function of complexity. Tissue complexity is measured as the number of cell types each human tissue has. Percentage disorder values of the proteins were predicted by IUPred. PPIs were taken from STRING, and only those pairs were counted where both partners were specifically annotated as expressed in that tissue by Swissprot. **(b) **Total number of binding sites as predicted by ANCHOR and total number of PPIs recorded by STRING in various human tissues as a function of tissue complexity. The fitted curves are linear but appear curved because of the log scale of the y-axis. **(c) **Median disorder in the main isoforms and the alternative complement as a function of the fraction of alternatively spliced (AS) tissue-specific proteins in Swissprot.

Figure 6a shows the relationships between the complexity of different human tissues (measured in cell types) and the median number of interacting proteins (derived from STRING, and restricted to those pairs that are both expressed in the tissue, according to Swissprot; blue squares) and also between the complexity and the median percentage disorder (as predicted by IUPred) of proteins expressed in that tissue (red dots). (As both interacting partners and disorder values were in the range 0 to 20, we conveniently used one scale for the two types of values.) Both protein interactions and disorder correlated with tissue complexity significantly (r = 0.7286, *P*-value = 0.0021 and r = 0.6762, *P*-value = 0.0056, respectively).

When we plot the two attributes (disorder and interacting partners) against each other (Additional file 5a) and fit a power function onto the values, we find that testis, ovary and liver show the highest deviations from the fitted curve: liver has more interactions than expected, which is explicable by the high number of enzymes it has, most of them known to be entirely globular. Testis and ovary on the other hand had more disorder than expected from the curve, probably due to the appearance of new, 100% disordered protein families, and also to the decrease in the number of PPIs taking place among germline-specific proteins [35].

Figure 6b shows the total number of binding sites predicted by ANCHOR and also the total number of PPIs recorded in STRING as a function of human tissue complexity. The fitted curve is calculated using linear regression, although the scale on the y-axis is logarithmic and the fitted lines therefore appear curved. Correlations between tissue complexity and the total number of PPIs, and between complexity and total number of predicted binding sites are both significant (r = 0.6978, *P*-value = 0.0038 and r = 0.7839, *P*-value = 0.0005, respectively). Plotting the predicted binding sites against the observed PPIs in STRING (Additional file 5b) will result in a close to quadratic function (y = 74.46 × x^0.519^), with a Spearman correlation value of r = 0.9412.

As there is a close link between alternative splicing and structural disorder [18], it is also of interest to see how this relationship plays out in tissues of different complexity. The median disorder of human proteins specifically expressed in different tissues (as annotated in Swissprot) is shown in Figure 6c as a function of the ratio of the alternatively spliced proteins for each tissue. The disorder of the splice variants is also shown for each tissue. Although both relations are noisy (r = 0.5912, *P*-value = 0.0159 for correlation between the alternative splicing ratio and disorder of the main isoforms; r = 0.6294, *P*-value = 0.009 between alternative splicing ratio and disorder of the alternative isoforms), they both implicitly reflect a positive correlation between complexity and both alternative splicing and structural disorder in the different tissues.

## Discussion

As both organismal complexity and structural disorder increase significantly from prokaryotes to eukaryotes, it is reasonable to assume that the two are related across a wider scope of evolution, especially if complexity (measured by the number of distinct cell types an organism has) cannot be related even to such a basic measure as the number of protein-coding genes of an organism, as reflected in the G-value paradox.

However, a simple comparison of proteome sizes measured in amino acids made it clear that if we treat plants as a separate group, complexity does correlate with the information content of the proteome (Figure 1) and less closely but still significantly even with gene number (Additional file 1). This finding weakens the G-value paradox, which holds up only when we include plants, which diverge considerably from the general trend between proteome size and complexity for metazoans.

On the other hand, there is no correlation between complexity and disorder beyond the already known significant increase between prokaryotes and eukaryotes, despite the critical roles disorder plays in PPIs [12-14], assembly of large protein complexes [15], and multiple activities of proteins [16]. To prove this, we provided a statistically rigorous test by correlating structural disorder with complexity in 76 species. Whether we look at the disorder of individual proteomes (Figure 2a) or large evolutionary groups (clades; Figure 2b), it becomes clear that apart from the significant increase between bacteria and eukaryotes, there is no further systematic increase in disorder in the latter. This probably follows from the wide roles protein disorder play even in primitive organisms, reflecting their lifestyle and adaptation to environmental factors, just as much as functional density [23] and other evolution-related features.

While not on a proteomic level, disorder does increase over evolution in structural domain-containing proteins and correlates significantly with complexity (Figure 2c, blue triangles). This is somewhat paradoxical as structural domains are globular, but can be explained by the presence of inter-domain linker regions, which are usually disordered, and perhaps by other functional aspects associated with disorder, such as signaling and protein-binding capability. A further link between complexity and disorder is apparent in the higher disorder of proteins containing an expanding-type domain (Figure 2c, red circles) when compared to proteins containing any-type or non-expanding domains.

Of other protein features, significant correlation could be observed with predicted binding regions of proteins and also the observed number of alternative splice variants (Figures 4 and 5). Both these features suggest that organism complexity increases with increasing functional complexity of gene products, because they enable one gene to bind more partners and/or translate into several protein products with potentially different functions.

We also investigated a potential link between structural disorder and complexity in different human tissues. With regard to ranking tissues, the BRENDA compilation of cell types was our major source of information [36,37]. Using their extensive listings of cell types found in different tissues, we could establish a three-way relationship between tissue complexity, PPIs and disorder (Figure 6a; Additional file 5a).

Similarly, we could also establish a three-way relationship between tissue complexity, the total number of recorded PPIs and the total number of predicted disordered binding sites (Figure 6b; Additional file 5b). As the latter follows closely a quadratic relationship (Additional file 5b) with a high Spearman correlation (r = 0.9412), this also means that twice as many binding sites result in about four times more PPIs; that is, protein binding tends to be promiscuous rather than one-on-one, at least for disordered binding sites that ANCHOR can predict.

## Conclusions

We found that the G-value paradox, at least within the scope of the organisms we studied here, could not withstand scrutiny and remained valid only when we grouped metazoans and plants together, as the gene number, and especially the total number of amino acids in all proteins, tends to increase with the complexity of the organism. It has also become clear that complexity is a multi-parametric trait that has many components at the protein level. We conclude that proteome size, structural disorder, alternative splicing and protein binding capacity all contribute to it, albeit to various extents, providing a finely tuned network that will enable an organism carry out its functions properly on all levels of its complexity.

## Materials and methods

### Proteome sequences

Out of almost 2,000 species with sequenced genomes (approximately 150 eukaryotes; from the Genome OnLine Database [38]), we selected 76 for this study for which the measure of complexity is reported in the literature [1-4]. Among these, 23 bacteria were selected to cover the full range of gene numbers ranging from 450 to 8,000; 53 eukaryotes were selected as in [2] complete with fully sequenced plant and other eukaryotic species to try to cover the full range of complexity, with gene numbers ranging from 3,800 to 92,000. The species and their number of different cell types (that is, complexity) are listed in Additional file 3. Sequences of prokaryotes were downloaded from the Expasy Proteomics Server [39]. The proteomes of eukaryotes were downloaded from Ensembl [40] and the National Center for Biotechnology Information [41]. If several splicing variants were present, we selected the longest transcript, except for the alternative splicing studies, where we considered them all.

### Prediction of structural disorder

Structural disorder of all proteins in a proteome was predicted with the IUPred algorithm [25], available at [42]. A residue was classified as locally disordered if its score was above the threshold of 0.5, and disorder of a protein was taken as the percentage of such residues in the protein. The average disorder of whole proteomes was calculated as the mean of the percentage of disordered residues of the proteins.

### Analysis of disorder of proteins with SCOP domains

The entire SCOP domain database was downloaded from [43] (release 1.75 [44]), and domain sequences were downloaded from the ASTRAL database [45,46]. We selected all domains belonging to superfamilies the expansion of which showed either good (Pearson correlation coefficient R ≥ 0.8) or poor (R ≤ 0.2) correlation with biological complexity, respectively, as reported in [2]. This resulted in three sets of protein sequences: expanding (containing a SCOP domain showing good correlation, *N *= 19,326), non-expanding (with SCOP domains showing poor correlation, *N *= 32,990) and all (containing any type of SCOP domains, *N *= 110,800). We ran a BlastP search with sequences of SCOP domains in the three sets against studied prokaryotic and eukaryotic proteomes. Then, we predicted structural disorder of proteins with sequence identity to a SCOP domain above 50%.

### Protein-protein interactions in different proteomes

We extracted data on binary PPIs in four prokaryotic (*Mycoplasma genitalium*, *Neisseria meningitidis*, *Escherichia coli*, *Streptomyces coelicolor*) and 12 eukaryotic proteomes (*Dictyostelium discoideum*, *Schizosaccharomyces pombe*, *Saccharomyces cerevisiae*, *Arabidopsis thaliana*, *Caenorhabditis elegans*, *Drosophila melanogaster*, *Ciona intestinalis*, *Danio rerio*, *Xenopus tropicalis*, *Gallus gallus*, *Mus musculus *and *Homo sapiens*) from the STRING database [47,48].

### Prediction of binding regions of proteins

Protein binding regions in disordered proteins/segments were predicted by the ANCHOR algorithm [20]. A binding site was predicted if there were at least three consecutive amino acids with a score above 0.5. Two adjacent binding sites were accepted as independent if they were separated by at least three residues with scores below 0.5. From the pattern of protein binding regions various measures were calculated, such as the average percentage of binding site residues in the proteome, the average number of binding sites per protein, and the total number of binding sites in the whole proteome.

### Determining the complexity of human tissues

The complexity of human tissues was determined by extracting cell type information for each tissue compiled by BRENDA [37] for the OBO Foundry [36], a coordinated effort to develop open biological and biomedical ontologies.

### Alternative splicing in different species and human tissues

We extracted alternative splicing information from the ASAP II database [9,49], which contains alternative splicing data for 15 animal species. For our studies we used splicing information for *Caenorhabditis elegans*, *Anopheles gambiae*, *Drosophila melanogaster*, *Danio rerio*, *Xenopus tropicalis*, *Gallus gallus*, *Bos taurus*, *Rattus norvegicus*, *Mus musculus*, and *Homo sapiens*. Various measures were calculated for each proteome, such as the percentage of alternatively spliced multi-exon genes and the average number of alternative splice events per protein. For human tissues we also extracted the available alternative splicing information from the Swissprot subset of Uniprot [50]. In addition, we extracted tissue-specificity information from the comment lines of the annotation starting with "CC" in Uniprot, wherever this information was available.

### Statistical analysis and programming

For calculating standard deviation values of intrinsic disorder and protein binding regions, random sampling was used. We selected random subsets of 200 to 500 members depending on the proteome size of the original dataset, and calculated the median and/or mean of disorder or number of binding regions. We repeated the selection 500 to 1,000 times, and the standard deviation of the mean was calculated. The significance of differences between selected groups was assessed by the nonparametric Mann-Whitney test. For correlation analysis we used a nonparametric (Spearman) test and chose two-tailed *P*-values, except for in Figure 1, where we assumed a Gaussian distribution for proteome size and calculated the Pearson correlation coefficients. All programs were written in Perl. The software IUPred [25] and ANCHOR [20] were obtained from the authors and were compiled and executed locally.

## Abbreviations

PIC: proteome information content; PPI: protein-protein interaction; SCOP: Structural Classification of Proteins.

## Competing interests

The authors declare that they have no competing interests.

## Authors' contributions

PT conceived and designed the experiments and wrote the paper. HH conceived and designed the experiments, performed the experiments, analyzed the data and wrote the paper. ES performed the experiments, analyzed the data and wrote the paper. All authors have read and approved the manuscript for publication.

## Supplementary Material

Additional file 1 **Correlation between gene number and complexity in 53 eukaryotic organisms**. Protein-coding gene numbers in all eukaryotes in the study excluding plants (red diamonds) and all plants (green triangles). Click here for file

Additional file 2 **Total mean proteome information content values for the six phylogenetic groups of the 76 species we used in this study**. Total mean proteome information content (PIC; measured as total number of amino acids contained in the main isoforms for each organism) values for the six phylogenetic groups of the 76 species we used in this study. After excluding plants we carried out a one-way analysis of variance among the remaining five groups, assuming Gaussian distribution for proteome sizes and used Bonferroni's multiple comparison test to check the significance of pairwise differences among the five phylogenetic clades. Click here for file

Additional file 3 **Prokaryotic and eukaryotic species used in our analysis, their reduced proteome size and their biological complexity**. This table also contains data related to disorder, binary protein-protein interactions, protein binding sites located in disordered regions of proteins and alternative splicing. Click here for file

Additional file 4 **Data related to disorder of proteins including SCOP domains in different prokaryotes and eukaryotes**. Click here for file

Additional file 5 **Disorder, protein-protein interactions and binding of proteins expressed in different human tissues**. Disorder, protein-protein interactions and binding of proteins expressed in different human tissues. **(a) **Median disorder of proteins versus median number of interacting partners in STRING. **(b)**Ttotal number of predicted binding sites versus total number of protein-protein interactions. Click here for file
