# Supplementary material for: The relationship between proteome size, structural disorder and organism complexity
Source: Genome Biol. 2011 Dec 19;12(12):R120. doi: 10.1186/gb-2011-12-12-r120 (PMC3334615; doi:10.1186/gb-2011-12-12-r120)
Supplement: Additional file 1 — Correlation between gene number and complexity in 53 eukaryotic organisms. Protein-coding gene numbers in all eukaryotes in the study excluding plants (red diamonds) and all plants (green triangles). [file gb-2011-12-12-r120-S1.PDF]

Suppl Figure 1. Pearson Correlation (gene number vs complexity)

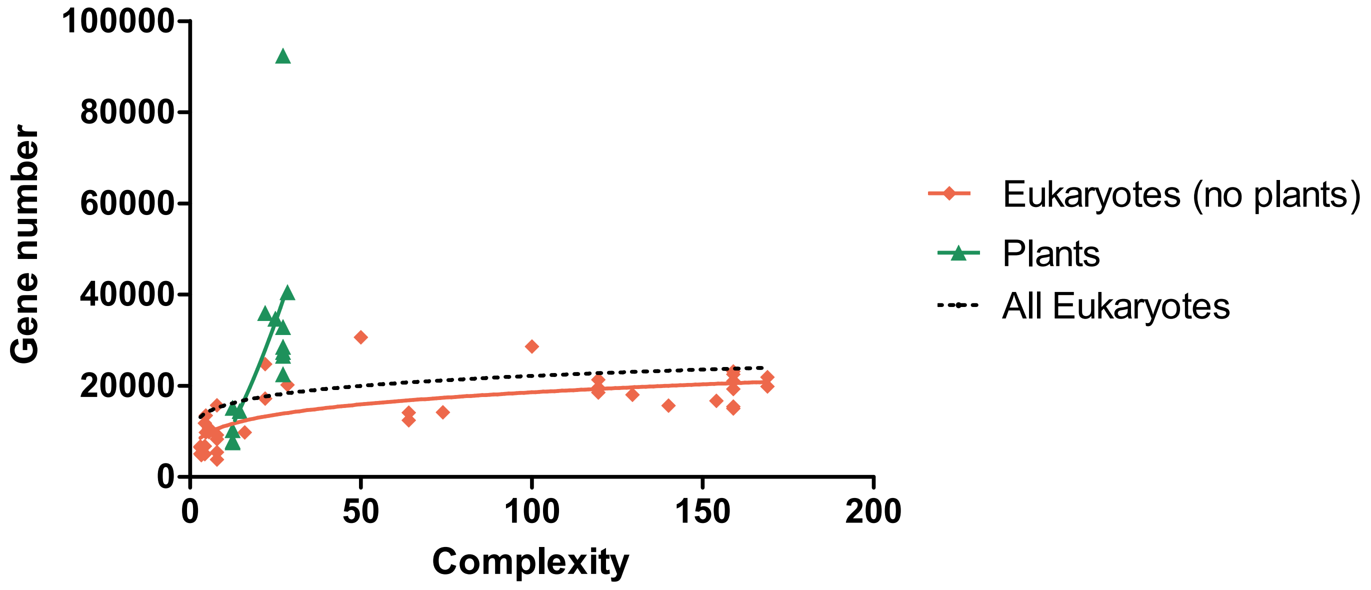

	Eukaryotes (no plants)	Plants	All Eukaryotes
Number of XY Pairs	39	14	53
Pearson r	0.6383	0.6223	0.1403
95% confidence interval	0.4042 to 0.7940	0.1367 to 0.8667	-0.1352 to 0.3957
P value (two-tailed)	P<0.0001	0.0175	0.3163
P value summary	***	*	ns
Is the correlation significant? (alpha=0.05)	Yes	Yes	No
R squared	0.4075	0.3872	0.01969
