# Supplementary material for: The relationship between proteome size, structural disorder and organism complexity
Source: Genome Biol. 2011 Dec 19;12(12):R120. doi: 10.1186/gb-2011-12-12-r120 (PMC3334615; doi:10.1186/gb-2011-12-12-r120)
Supplement: Additional file 2 — Total mean proteome information content values for the six phylogenetic groups of the 76 species we used in this study. Total mean proteome information content (PIC; measured as total number of amino acids contained in the main isoforms for each organism) values for the six phylogenetic groups of the 76 species we used in this study. After excluding plants we carried out a one-way analysis of variance among the remaining five groups, assuming Gaussian distribution for proteome sizes and used Bonferroni's multiple comparison test to check the significance of pairwise differences among the five phylogenetic clades. [file gb-2011-12-12-r120-S2.PDF]

Supplementary Figure 2

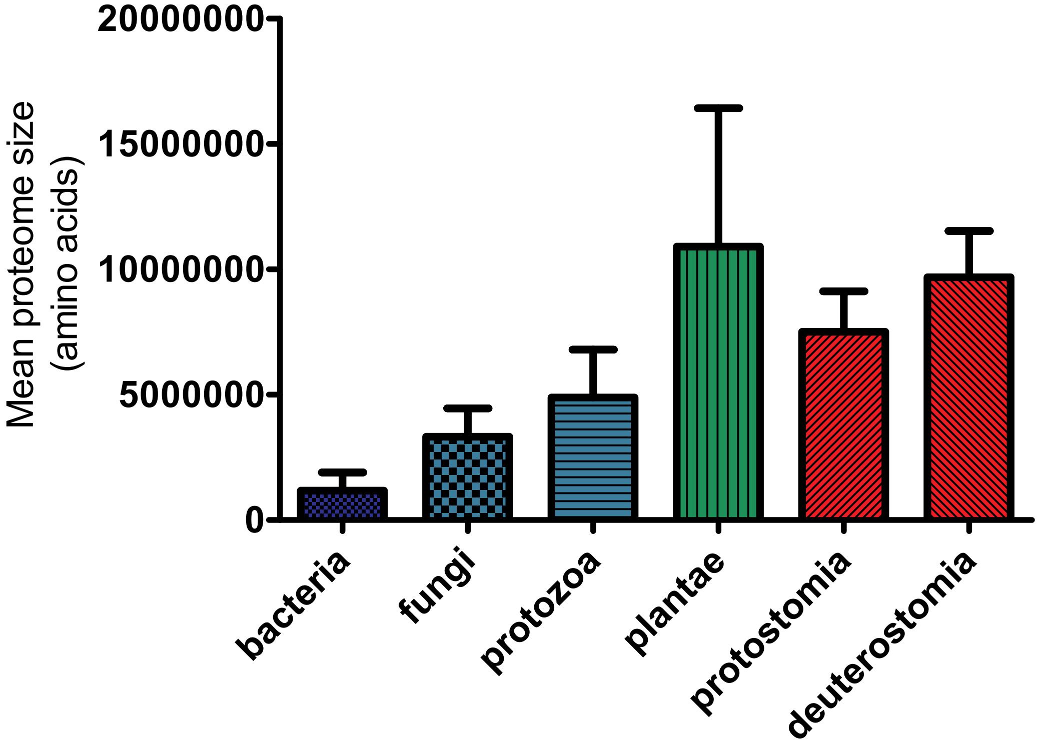

Table Analyzed	5 phylogenetic groups, plants excluded
One-way analysis of variance	
P value	P<0.0001
P value summary	***
Are means signif. different? (P < 0.05)	Yes
Number of groups	5
F	97.22
R squared	0.8722

Bonferroni's Multiple Comparison Test	Mean Diff.	t	Significant? P < 0.05?	Summary
bacteria vs fungi	-2141000	3.912	Yes	**
bacteria vs protozoa	-3715000	6.182	Yes	***
bacteria vs protostomia	-6332000	10.53	Yes	***
bacteria vs deuterostomia	-8502000	18.76	Yes	***
fungi vs protozoa	-1574000	2.243	No	ns
fungi vs protostomia	-4190000	5.972	Yes	***
fungi vs deuterostomia	-6360000	10.96	Yes	***
protozoa vs protostomia	-2616000	3.515	Yes	**
protozoa vs deuterostomia	-4787000	7.586	Yes	***
protostomia vs deuterostomia	-2170000	3.440	Yes	*
