# Supplementary figures and images for: The relationship between proteome size, structural disorder and organism complexity
Source: Genome Biol. 2011 Dec 19;12(12):R120. doi: 10.1186/gb-2011-12-12-r120 (PMC3334615; doi:10.1186/gb-2011-12-12-r120)

## Supplementary Figure 3A

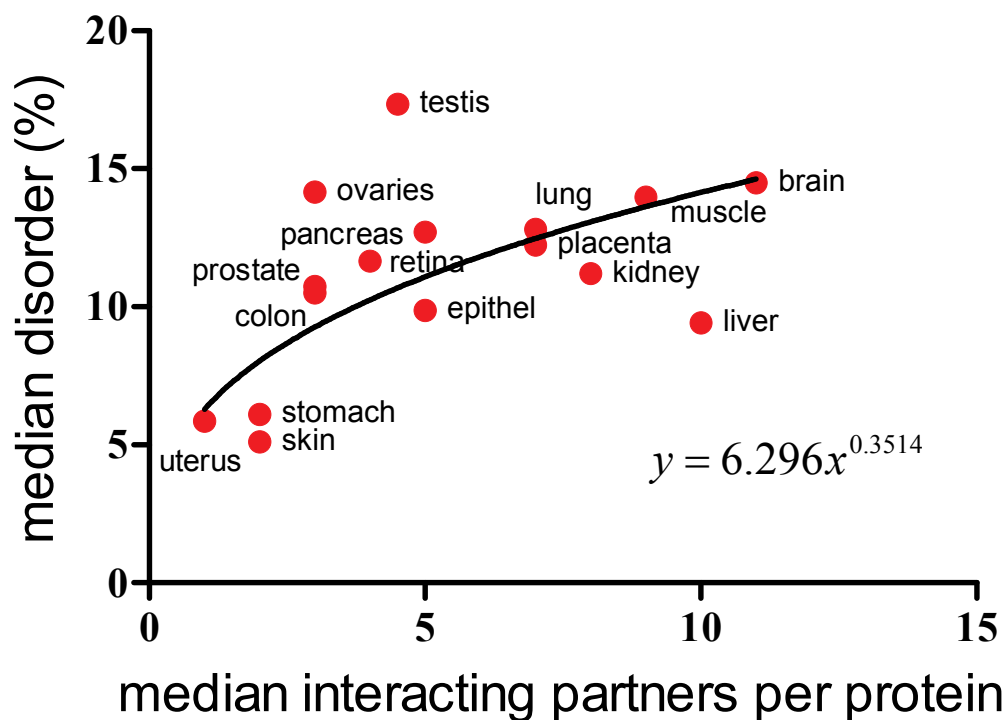

## Supplementary Figure 3B

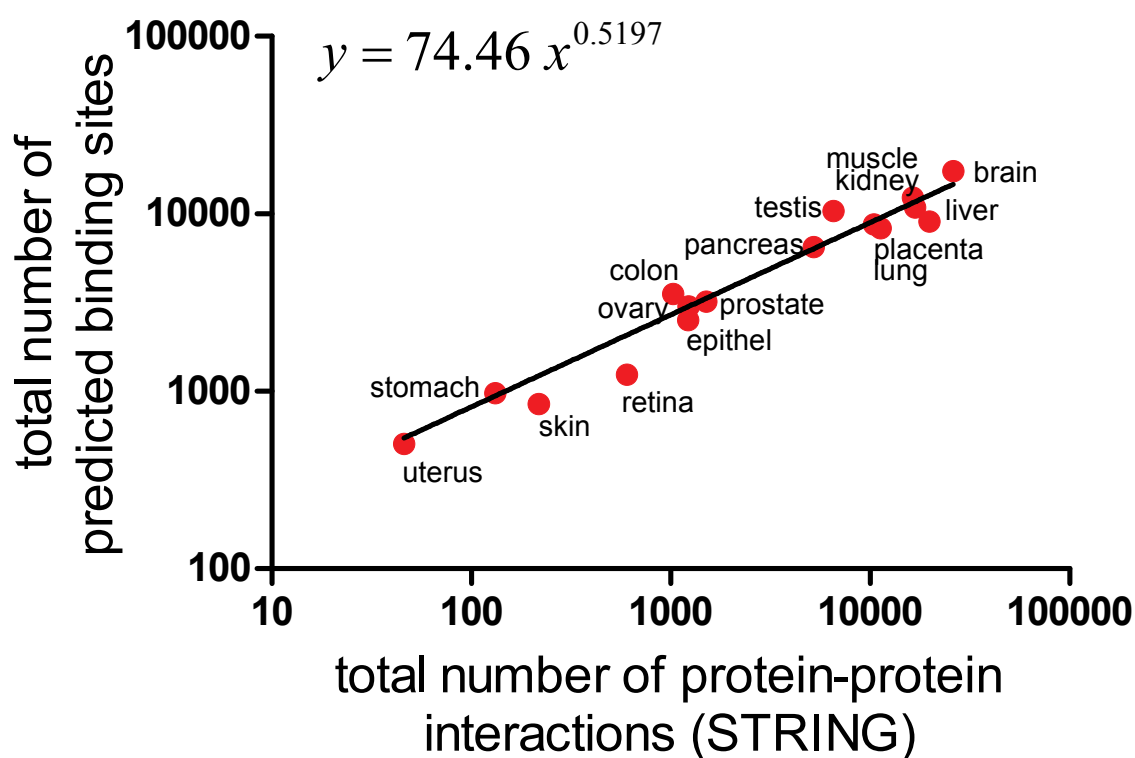

Supplement: Additional file 5 — Disorder, protein-protein interactions and binding of proteins expressed in different human tissues. Disorder, protein-protein interactions and binding of proteins expressed in different human tissues. (a) Median disorder of proteins versus median number of interacting partners in STRING. (b)Ttotal number of predicted binding sites versus total number of protein-protein interactions. [file gb-2011-12-12-r120-S5.PDF]
